# Trends of seroprevalence of Chagas diseases in healthy blood donors, solid organ donors and heart transplant recipients: experience of a single health care centre in Colombia

**DOI:** 10.1017/S0950268820002721

**Published:** 2020-11-06

**Authors:** María Elena Tello-Cajiao, Olga Lucia Agudelo-Rojas, Marcela Quintero, Laura Cardenas, Fernando Rosso

**Affiliations:** 1 Centro de Investigaciones Clínicas (CIC), Fundación Valle del Lili, Cali, Valle del Cauca, Colombia; 2 Blood Bank, Fundación Valle del Lili, Cali, Valle del Cauca, Colombia; 3 Fundacion Valle del Lili, Internal Medicine Department, Infectious Disease Service, Cali, Valle del Cauca, Colombia

**Keywords:** Blood donors, Chagas disease, organ transplantation, *Trypanosoma cruzi*

## Abstract

The prevalence of Chagas disease has decreased in the Americas region due to vector control measures. However, non-vectorial transmission through blood transfusions and organ transplantation has gained importance in recent years. Screening among blood and organ donors are essential to reduce *Trypanosoma cruzi* transmission and could provide information to estimate population prevalence. We conducted a cross-sectional study on the prevalence of immunoglobulin G (IgG) antibodies against *T. cruzi* in healthy blood donors, solid organ donors and heart transplant recipients from 2012 to 2019. We found a total of 99 357 IgG *T. cruzi* results during the study period. The cumulative seroprevalence in healthy blood donors was 0.13% (95% confidence interval (CI) 0.10–0.15), in organ donors was 0.53% (95% CI 0.06–1.92) and in heart transplant recipients was 3.03 (95% CI 0.07–15.75). Seroprevalence trend in healthy blood donors showed annual increase between 2012 and 2015, decreasing in the following years. No trend was seen in organ donors neither heart recipients. Adjusted rates did not show difference by sex and age among blood donors. No significant increases in seroprevalence *T. cruzi* were found during the study period. *T. cruzi* transmission remains low.

## Author summary

The prevalence of Chagas disease has decreased in the Americas region due to vector control measures. However, transmission through blood transfusions and organ transplants has gained visibility due to the migratory phenomena of asymptomatic carriers of the parasite, which have increased cases in non-endemic areas. In this study, the authors describe the seroprevalence of Chagas infection in blood donors, solid organ donors and cardiac recipients over 7 years. It was found that, although the seroprevalence of *Trypanosoma cruzi* infection remains low, it is important to detect people with undetermined forms of the infection through these mechanisms in order to do secondary prevention. Especially, because any person could become a blood donor or need an organ transplant, thus knowing the behaviour of seroprevalence in endemic countries, provides a broader picture of the importance of epidemiological surveillance of the infection. Screening measures should continue in the context of transfusion and organ transplantation safety, because a single case of infection represents a considerable disease burden for health systems and individuals.

## Introduction

American Trypanosomiasis or Chagas disease is a parasitic infection caused by the protozoan *Trypanosoma cruzi*. About 70 million people worldwide are at risk of acquiring it and it is estimated that 7 million are already infected. The Americas region has the highest concentration of cases, especially in countries such as Colombia, where it is endemic [1–4]. Although in recent years the infection prevalence in the country has remained close to 1% thanks to vector control measures, it is considered to be re-emerging due to the constant migration of individuals who develop indeterminate forms of the infection and serve as parasite reservoirs [5–8].

In this sense, transmission through organ transplantation and blood donation is highly feasible. In fact, transmission through the donation of blood products from asymptomatic carriers represents the second most frequent way of acquiring the parasite (20%) [6, 9]. In 2017, according to National Blood Bank Network, the reactivity of *T. cruzi* in donated whole blood units was 0.19% in the country [10]. Despite having a low prevalence, the cumulative number of Chagas disease cases reported to the National Health Institute (INS – Spanish acronym) in 2019 was 486 (250 chronic and 236 acute). Of these, 182 chronic cases (72.8%) and 53 acute infections (22.5%) were confirmed, mainly in groups in the northeastern departments of the country [11, 12]. Therefore, blood banks are an important source of detection of chronic cases [13]. Although in organ transplantation there is a documented risk of reactivation in the recipient of an infected organ, facilitated by the immunosuppressive therapy required in the post-transplant stage [14, 15].

Given the constant presence of chronic cases in the country and the possible risk of transmission by blood donation and organ transplantation, an observational study was conducted to describe the seroprevalence of *T. cruzi* infection in both, blood donors and donors and organs recipients, over a period of 8 years.

## Methods

### Design, population and data collection

A cross-sectional descriptive study was carried out between January 2012 and October 2019 at the Fundación Valle del Lili University Hospital (FVL), a national leading institute in organ transplant. Serum samples from volunteer blood donors, solid organ donors and heart transplant recipients were included in the study. We selected patients of both sex, who had undergone an immunoglobulin G test (IgG) for *T. cruzi* (Chagas) infection. Patients without documented test results were excluded. The data were obtained from the blood bank, the institutional transplant registry and the microbiology laboratory. A double check over 10% of the records was done in order to verify quality of the sources. This research was reviewed and approved by the FVL Biomedical Research Ethics Committee.

### Variables of interest

The study variables were: type of event (blood donation, organ donation or heart transplant recipient), age, sex, origin, year of donation or transplant and serological result of a Micro-Particle Chemiluminescence Immunoassay (MPCIA) platform Architect System (Abbott). Positive results were processed in duplicate. Double-positive samples were confirmed by indirect immunofluorescence (IFI) until June 2018. For confirmation of positive cases after this date, the samples sent to a public health laboratory were subjected to enzyme-linked immunosorbent assay (ELISA) test. The outcomes evaluated were the annual and cumulative seroprevalence according to the type of donation, adjusted by sex and age.

### Statistical analysis

The proportion of IgG seroprevalence was calculated according to the type of subject: blood donor, organ donor and organ recipient, and compared using a *Z* test. For the calculation of crude seroprevalences in blood donors, the laboratory provided pooled data of the number of donations per year and the number of IgG-positive sera independent of the confirmatory infection result.

To describe Chagas IgG seroprevalence data in blood donors, absolute and relative frequencies were calculated, with the total donor population per year as the denominator. The seroprevalence trend over time was analysed using a simple linear regression line. Odds ratios (OR) of seroprevalence per year were estimated, taking the first year of the series as a reference. Data are shown in Figure 3. Finally, crude and adjusted IgG seroprevalence rates were calculated by a direct method, taking the sum of total male and female blood donors as the standard population, in time and age strata. All calculations were performed using 95% confidence intervals (95% CI) and taking values of *P* < 0.05 as significant. The analyses were performed using Stata Corp 14^®^ software.

## Results

**Fig. 1.**
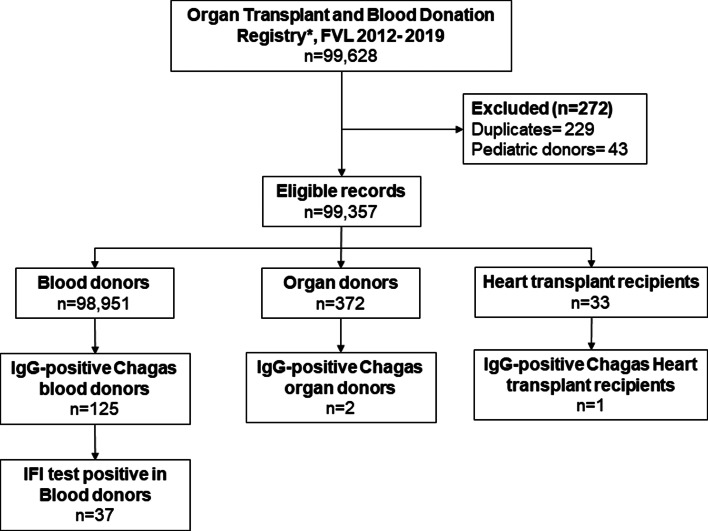
Flowchart of data management.

From the institutional registry on blood donors, we had access to pooled data of 7 years (2012–2019) of results of IgG antibodies against *T. cruzi* results of the IFI test and ELISA test results, by age group and sex. From the institutional registry on organ transplantation, we identified 677 patients with documented IgG Chagas serological test results in the study period, from which 229 duplicate records and 43 paediatric donor records were excluded. Finally, data were pooled for analysis, including information from 372 organ donors and 33 heart transplant recipients (Fig. 1).

**Fig. 2.**
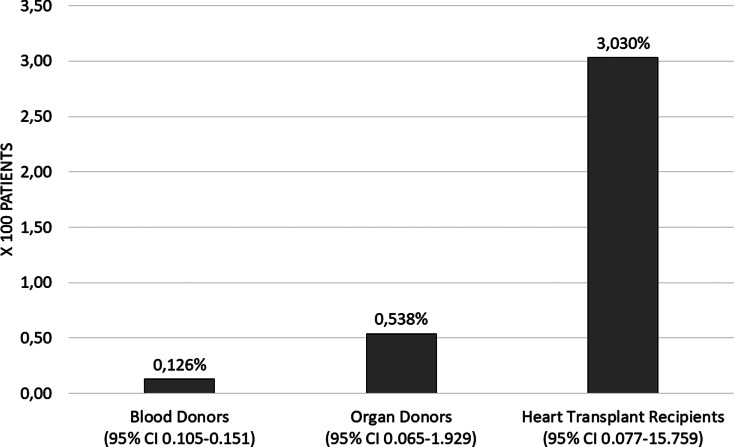
Proportion of seropositivity of IgG Chagas in recipients and donors, FVL 2012–2019. Heart transplant recipient data 2012–2016.

The proportion of people with positive Chagas IgG in blood donors during the study period was 0.126% (95% CI 0.105–0.151) corresponding to 125 cases. The proportion of organ donors was 0.53% (95% CI 0.065–1.929) corresponding to two cases and the proportion of heart transplant recipients was 3.03 (95% CI 0.077–15.759) corresponding to one case (Fig. 2). The proportion of IgG Chagas seropositivity in blood donors was statistically lower compared to the proportions in organ donors and recipients (*P* = 0.003). The proportion calculated for cardiac recipients was the highest (*P* = 0.029).

**Fig. 3.**
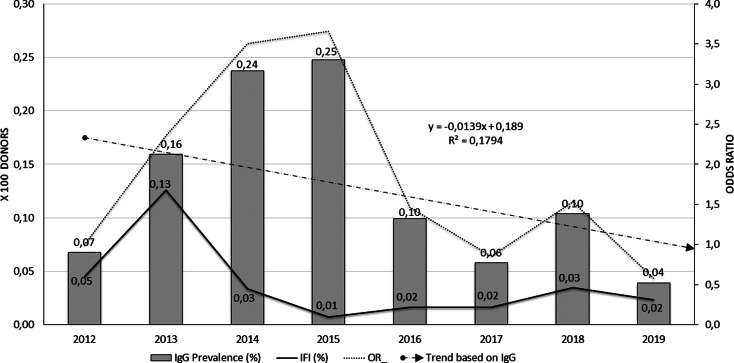
Behaviour of seroprevalence and confirmation of Chagas cases in blood donors, FVL, 2012–2019.

The analysis of Chagas IgG seroprevalence and confirmed cases in blood donors can be seen in Figure 3. The grey bars show the behaviour of Chagas IgG seropositivity in blood donors over the studied years; they highlight the increased proportion of seropositive between 2013 and 2015, with an observed frequency of 25 cases per 10 000 donors (0.25%) in 2015 and a decrease in the following years.

The proportion of confirmed cases (continuous black line) was significantly higher during 2013 (13 cases per 10 000 donors), remaining low for the next years of the series. Although 2018 shows a slight increase in seroprevalence, in general, the frequency of the event was low in the accumulated years (0.04%). The trend line of seroprevalence line (cut line) models the decrease if the event through time, but with low explanatory capacity (*R*^2^ = 0.17). No trend was seen in organ donors or heart recipients.

The unadjusted opportunity of having a positive Chagas IgG result among blood donors in the cumulative years increased significantly compared to the baseline year 2012 (OR 2.0, 95% CI 1.02–4.49, *P* = 0.04). However, the strata of the years variable show that the greatest risk was at the beginning of the series, with the year 2015 presenting the greatest opportunity to present positive IgG for Chagas among blood donors (OR 3.66, 95% CI 1.71–8.69, *P* = 0.0002). This behaviour is plotted by the dotted line of Figure 3. In the last 4 years, seroprevalence frequencies again resemble that observed in 2012, so the opportunity decreases from 2016 onwards with no significant differences between them.

The proportion of blood units donated by men in the study period was 51.67% (95% CI 51.4–51.9), whereas for women it was 48.3% (95% CI 48.02–48.6). The calculation of relative frequencies in seropositive blood donors did not show differences by sex and age; however, most of the individuals screened were of urban origin (99.2%). Similarly, the seroprevalence rates calculated to compare the population of male and female blood donors, standardised by age groups and years of study, did not show relevant differences either (Table 1). In the case of organ donors, a 52-year-old man (2017) and a 39-year-old woman (2017), both of urban origin, were found. The recipient of the heart transplant was a 47-year-old man (2013) from an urban area.

**Table 1.** IgG Chagas seropositivity rates in women and men blood donors, adjusted by year and age groups (*n* = 125)

Rate/sex	Female, *n* = 63	Male, *n* = 62
	% (95% CI)	% (95% CI)
Crude rate	0.132 (0.101–0.169)	0.121 (0.093–0.155)
Adjusted rate per year*	0.129 (0.108–0.154)	0.120 (0.100–0.144)
Age-adjusted rate	0.137 (0.115–0.163)	0.116 (0.096–0.139)

* 2012–2019 means that the study was carried out between 2012 and 2019.

The standardised ratio of rates by age group was 1.07 cases in women for each case in men, whereas by age group it was 1.18 cases in women for each case in men. No differences in sex and age distribution were found in organ donors or heart transplant recipients, given the very low number of cases. Nor was information obtained on documented confirmation tests in either of these two groups. However, the positive case in the recipient group corresponded to a heart transplant from a patient previously diagnosed with long-standing Chagasic heart disease.

## Discussion

This study described the trend of seroprevalence of Chagas infection in blood donors, solid organ donors and heart transplant recipients across 8 years. According to the findings, the seroprevalence in blood donors was the lowest among the study subjects (0.126%), with no differences by sex or age. The analysis by year concluded that both, the frequency and relative opportunity of finding reactive blood donors, tend to decrease over time as do confirmed cases. Meanwhile, the seroprevalence in organ donors and heart transplant recipients was 0.53% and 3.03%, respectively.

According to the National Network of Blood Banks reports, local seroreactivity in blood donors remains close to 0.13% [10], and below that expected for the endemic areas of Central and South America, whose prevalence is 0.2% [5]. A summary of other studies in blood donors from different regions and years is presented in Table 2. Most of them show similar seroprevalences and sociodemographic characteristics to this study, except for the study performed by Remesar *et al*. in the region known as Chaco, which is highly endemic [16–24].

**Table 2.** Summary of prevalence of *T. cruzi* infection among blood donors in previous studies

Authors	Number of donors tested	Age	Gender	Seroprevalence in blood donors (%)	Years of study
Present study (2020), Colombia	98 951	No differences among age ranges	No differences among males and females	0.126	7
González-Guzmán *et al*. [16], Mexico	510 047	Higher among 51–65 years	Higher in males	0.12	8
Miranda *et al*. [19], Brazil	494 010	Higher in >30 years	Higher in males	0.62	10
Sayama *et al*. [18], Japan	18 484	Median was 35 years	Higher in males	0.017	12
Santana *et al*. [21], Brazil	170 148	Higher in >44 years	Higher in males	0.010	9
Remesar *et al*. [22], Argentina	1423	Mean was 40.6 years	No differences among males and females	21.4	1
Borelli *et al*. [23], Brazil	8337	Higher among 33–43 years	No differences among males and females	0.02	1
Zaniello *et al*. [17], USA	876 614	Median was 43 years	Higher in females	0.0079	3
El Ghouzzi *et al*. [24], France	30 837	Mean 34 years	No differences among males and females	0.01	1.5
Piron *et al*. [20], Spain	1770	Mean was 35 years	Higher in males	0.65	1

In organ transplantation, there are several published case reports of the infection in both donors and recipients, but few available studies of cumulative seroprevalence in this population. One of them is the study by da Costa *et al*. in Brazil, who found a seroprevalence in potential organ donors of 1.3%, which was different from ours [25]. It is also important to assess the risk of reactivation of the infection in the post-transplant stage. Some authors have shown that this risk varies according to the anatomical piece and the degree of immunosuppression to which the patient is subjected. For example, the risk of reactivation for kidney, liver and bone marrow receptors has been reported between 8% and 27%, whereas for heart transplants it can be up to 50%, due to the parasite's tropism with this tissue [2, 15, 25]. In this study, there was no information on reactivation of the infection in the analysed patients.

With regards to the population, the frequency of the blood component donation in men was found to be significantly higher (51.6%), but there was no distinction in the cumulative reactivity rates for *T. cruzi* adjusted for sex or age. However, several studies have shown a higher frequency of infection in men aged 25–49 years, not only in general population serosurveys but also in reactivity studies in blood donors. This suggests that there seems to be a differential risk that some authors have explained from an occupational point of view [13, 25–27]. As for women of childbearing age, the collection of reactive sera is very timely in preventing congenital disease, taking into account that this represents up to 5% of all forms of transmission [2, 6].

It seems that the higher seroprevalence found in units of blood screened at the beginning of the series and the subsequent decrease in the probability of infection over time (Fig. 2), can be explained by the frequent extramural donation campaigns that were carried out in the early years; a situation that would have attracted a greater number of people from peri-urban areas. However, improvements in transfusion safety practices are also attributable to increased screening coverage of blood units and more rigorous donor selection.

As limitations, it can be said that the seroprevalence found in this study does not represent the real risk of transmission by these mechanisms, but confirms the importance of continuing to screen infection in organ transplant and blood donation. It is to be considered that 70–80% of those infected will develop indeterminate forms of the disease and will behave as reservoirs of the parasite [7, 28]. In addition, in the complex context of a transplant, complete information on epidemiological history may not be available at the time of donation, especially in the case of post-mortem donors. Another identified limitation is that it was not possible to differentiate seroprevalence in whole blood from that estimated by apheresis. It is important because the seroprevalence of infection reported by apheresis is usually higher and could imply a differential risk [10]. Also, more information about sociodemographic conditions of the donors and recipients could be collected in future studies, to get a better characterisation of the population.

## Conclusions

Although the seroprevalence of infection was low, detection strategies should be continued given the presence of chronic carriers who can become donors (of blood or organs) or recipients of organs throughout their lives. Furthermore, it is important to detect people with asymptomatic infection through these mechanisms, to make secondary prevention and predict the potential reactivation of the infection in immunosuppressed patients, like those who undergo organ transplants.

## Data Availability

The data that support the findings of this study are available from the corresponding author, upon reasonable request.
